# Recent progress in the intranasal PLGA-based drug delivery for neurodegenerative diseases treatment

**DOI:** 10.22038/IJBMS.2023.70192.15264

**Published:** 2023

**Authors:** Nasim Aghaei Delche, Reyhaneh Kheiri, Behnam Ghorbani Nejad, Mojgan Sheikhi, Malihe Sadat Razavi, Milad Rahimzadegan, Zahra Salmasi

**Affiliations:** 1 Department of Biology, Payame Noor University, Tehran, Iran; 2 Department of Pharmacology and Toxicology, Faculty of Pharmacy, Tehran University of Medical Sciences, Tehran, Iran; 3 Department of Toxicology and Pharmacology, Faculty of Pharmacy, Kerman University of Medical Sciences, Kerman, Iran; 4 Department of Drug and Food Control, Faculty of Pharmacy, Tehran University of Medical Sciences, Tehran, Iran; 5 Department of Pharmaceutics, Faculty of Pharmacy, Mazandaran University of Medical Sciences, Sari, Iran; 6 Nanotechnology Research Centre, Faculty of Pharmacy, Tehran University of Medical Sciences, Tehran, Iran; 7 Functional Neurosurgery Research Center, Shohada Tajrish Comprehensive Neurosurgical Center of Excellence, Shahid Beheshti University of Medical Sciences, Tehran, Iran; 8 Nanotechnology Research Center, Pharmaceutical Technology Institute, Mashhad University of Medical Sciences, Mashhad, Iran

**Keywords:** Drug delivery, Intranasal, Nanoparticle, Neurodegenerative diseases, PLGA

## Abstract

One of the most challenging problems of the current treatments of neurodegenerative diseases is related to the permeation and access of most therapeutic agents to the central nervous system (CNS), prevented by the blood-brain barrier (BBB). Recently, intranasal (IN) delivery has opened new prospects because it directly delivers drugs for neurological diseases into the brain via the olfactory route. Recently, PLGA-based nanocarriers have attracted a lot of interest for IN delivery of drugs. This review gathered clear and concise statements of the recent progress of the various developed PLGA-based nanocarriers for IN drug delivery in brain diseases including Alzheimer’s, Parkinson’s, brain tumors, ischemia, epilepsy, depression, and schizophrenia. Subsequently, future perspectives and challenges of PLGA-based IN administration are discussed briefly.

## Introduction

***Nose-to-brain drug delivery***

Since the olfactory mucosa became known as a window into the central nervous system (CNS) and brain, intranasal (IN) delivery has attracted the attention of scientists because of its ability to directly target neurological diseases (1, 2). This route does not have the limitations of common ways such as the blood-brain barrier (BBB), which can be promising in the treatment of neurodegenerative diseases (3, 4). 

**Figure 1:**
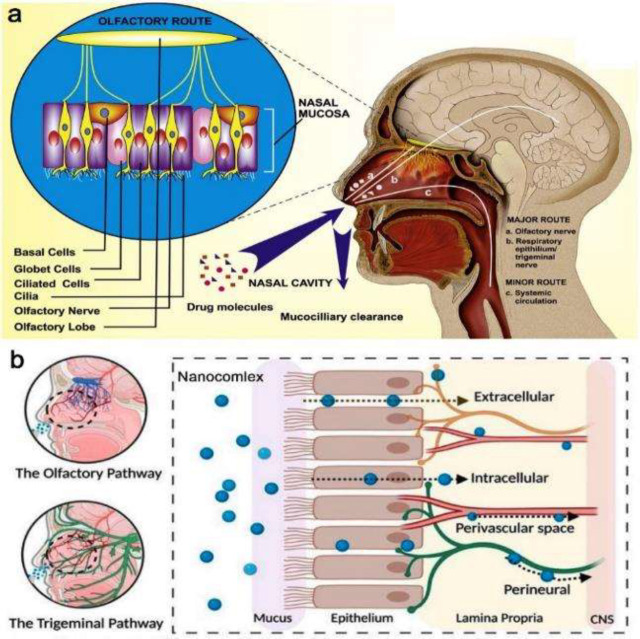
**. **a) Mechanism of nose-to-brain drug delivery through the olfactory, trigeminal, and systemic pathways, b) the nanocarrier distribution pathway after IN administration

Reprinted with permission from (6) and (21)

The systemic, olfactory, and trigeminal nerve pathways are the various routes that have been suggested for nose-to-brain drug delivery (Figure 1a). In a systemic-based pathway, the drug enter into the systemic circulation through direct absorption to the vascular nasal epithelial cells. This mechanism is a routine way for the transmission of low molecular weight therapeutic agents to the target site in the brain through the BBB (5, 6). Olfactory (nerve & epithelia) and trigeminal nerves are mechanisms for brain-targeted drug delivery. These pathways allow the rapid onset of action, decrease systemic exposure, and can circumvent the BBB (4). The main mechanism for the nose-to-brain drug delivery of drugs absorbed from the olfactory region is the vascular and nerve fibers pathway. The drugs internalize into the olfactory neurons by pinocytosis and endocytosis and then transport into the olfactory bulb, followed by distribution to different sites of the brain (5, 7).

Recently, nanoscale vehicles represented the new potential for targeted drug delivery via the olfactory route (8). Drug-loaded nanocarriers provide numerous advantages for nose-to-brain delivery such as better reception by more patients compared with injection, overcoming biological barriers such as first-pass metabolism, intestinal degradation, BBB, rapid drug absorption, and enhanced bioavailability (5, 6). Among drug delivery systems, Poly(lactic-co-glycolic acid) (PLGA) NPs are considered promising transfer systems due to their unique properties (9).

***PLGA***

Synthetic polymers such as PLGA have been extensively used as colloidal materials for the production of nanoparticles (NPs)-based nanocarriers for drug delivery (8, 10). Synthetic polymers have higher reproducibility and purity than natural polymers. PLGA is a well-known FDA-approved biodegradable copolymer consisting of lactic and glycolic acids (11, 12). PLGA is highly used in clinical applications especially in targeted drug delivery due to its biocompatibility and biodegradability properties (13). PLGA as a drug delivery nanocarrier is widely used as a vehicle for proteins, drugs, and various other molecules such as peptides, DNA, and RNA (14-16). By controlling the relevant parameters such as molecular weight of the polymer, lactide/glycolide ratio, and drug concentration, the overall physical properties of the polymer-drug matrix can be adjusted depending on the drug, desired dose, and release time (17, 18). 

The surface modification of the PLGA NPs by specific ligands such as aptamer, peptides, or other polymers such as polyethylene glycol (PEG) leads to a targeted colloidal nanocarrier, which can escape from the reticuloendothelial system and target the specific receptor in the treatment site (19, 20). In recent years, several PLGA-based nanocarriers have been prepared to deliver different therapeutic agents to the brain through the IN pathway (Figure 1b).

In the current review, the recent advances of the drug-loaded PLGA-based nanocarriers that have been designed for successful nose-to-brain drug delivery are discussed. In addition, the major considerations in the fabrication of PLGA-based nanocarriers for IN administration are reviewed. Finally, the drawbacks and challenges of nose-to-brain PLGA-based drug delivery to effectively treat brain diseases are also explained.

***Intranasal PLGA-based drug delivery for neurodegenerative disease treatment***

Due to diagnostic and therapeutic complications, neurodegenerative diseases face many problems. Numerous physiological barriers, such as intestinal barriers, nasal, dermal, and BBB, limit the use of drugs in the treatment of such diseases. Therefore, drug delivery *via* the nasal route provides potential for brain targeting. Unfortunately, the low amount of drugs accessing the brain from the nasal route is due to the nasal epithelial barrier. To overcome the nasal-related barrier, the administration of drugs by appropriate nanocarriers such as targeted PLGA can be proper. The uptake of PLGA-based NPs in olfactory cells has been confirmed by Rhodamine (RhB)-loaded NPs manner (22, 23).

***Alzheimer’s***

One of the most common types of neurodegenerative dementia in aging is Alzheimer’s disease (AD) (24). AD includes central neurodegenerative disorders, regardless of clinical condition. Pathological symptoms that characterize AD include progressive accumulation of senile plaques, synapse loss, and neuronal atrophy (1, 25). AD is determined by personality changes, progressive memory, and cognitive impairment, which results in dementia and death (19). Other symptoms of AD include impaired cognitive skills and memory, and a gradual loss of capacity to perform daily activities. Due to the increasing aging of the population around the world, AD has become one of the most important public health concerns. Biomarkers are one of the most reliable indicators to identify AD disease. Therefore, three biomarkers beta-amyloid (Aβ), apolipoprotein E4 (ApoE4), and Tau protein have been routinely used for the differential diagnosis of AD in cerebrospinal fluid (CSF) and blood (26). Recently, PLGA-based IN drug delivery is considered a novel strategy to treat AD disease (Table 1).

In this line, first, Mathew *et al.* used the encapsulated Curcumin (CUR) in PLGA NPs for the treatment of AD. CUR known as an anti-oxidant has exhibited high efficiency in oxidative stress in AD management (27). In this study, to overcome the passing problem through BBB, the NPs were coupled with Tet-1 peptide, which has possessed retrograde transportation properties and affinity to neurons. The results showed that the prepared formulation was non-toxic, retained its anti-oxidant effects, and can destroy Aβ masses (28). In a similar study, CUR and bisdemethoxycurcumin were encapsulated in PLGA NPs for targeted delivery to the brain through the nose. In this study, their therapeutic effects were confirmed on the inhibition of P-gp BBB efflux pumps and targets including cyclooxygenase2 (associated with inflammatory reactions) and Aβ masses (29).

**Figure 2:**
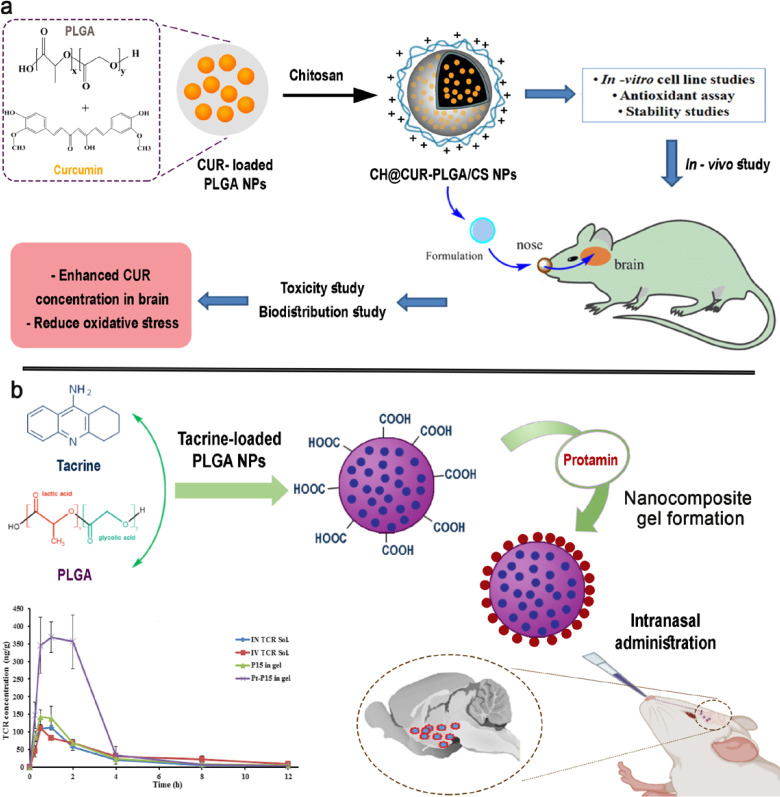
a) IN delivery of CUR/PLG A/CS, b) Pt/PLGA nanocarrier preparation and IN administration for AD treatment

In the same research, Zhang and coworkers fabricated CUR-loaded hydroxypropyl-β-cyclodextrin nanocarriers (CUR/HP-β-CD) and CUR-encapsulated chitosan (CS)-coated PLGA NPs (CUR-CS-PLGA), administered *via* the IN route, and compared. CS is a cationic mucoadhesive polymer, which enhances the residence time of NPs at the target site and improves the drug permeation. The results of *in vitro* studies revealed the high stability, cellular uptake, and cytotoxicity of CUR/HP-β-CD NPs in SH-SY5Y cells. In addition, both formulations could decrease IL-6 and TNF-α levels (40 and 70 %, respectively), which indicated the anti-inflammatory effects of both formulations (30). Other CUR/CS PLGA NPs were developed for IN administration and compared with free PLGA NPs (Figure 2a). The developed NPs were characterized by particle size in the range of 200 nm and CUR encapsulation with 75% entrapment efficiency (EE). Permeation and release study revealed enhanced permeation and sustained release through the nasal mucosa. Caveolae-mediated endocytosis of NPs were observed as a cellular uptake mechanism. The results also showed a higher bio-distribution of CUR/CS PLGA NPs through the IN route (31).

In a study, an IN strategy was developed based on cell-penetrating peptides (CPPs)-conjugated PLGA NPs for insulin delivery to the brain to assess its possible therapeutic effect on AD. The results showed the ability of CPPs in the nose-to-brain delivery of insulin, which is considered a potential treatment for neurodegenerative disorders (32). Forasmuch as lectins-modified PEG-PLGA NPs could increase brain drug delivery after IN application, in a study, basic fibroblast growth factor (bFGF)-loaded NPs modified with *Solanum tuberosum* (Potato) lectin (STL) were developed for IN brain delivery. STL can selectively bind to N-acetylglucosamine on the nasal epithelial membrane. The radioisotope tracing method for the assessment of brain distribution of the formulations revealed that the areas under the concentration-time curve of formulation in the cerebrum, cerebellum, and olfactory bulb of rats through IN administration were higher than that of intravenous inoculation. The neuroprotective effect of nanocarriers showed improved spatial learning and memory of AD rats in the STL-loaded PLGA-received group (33).

Some drugs may not be effective in treating AD due to their low penetration and low bioavailability into the brain. Tarenflurbil (Flurizan/TFB) is one of these drugs that failed due to low penetration in phase 3 clinical trials in AD patients. In this regard, TFB was used along with PLGA nanocarriers and solid lipid NPs (SLNs). The EE and release rates of TFB-PLGA and TFB/SLNs are relatively higher than the free form of the drug. Pharmacokinetic studies also showed the higher bioavailability, biodistribution, and drug targeting efficiency of TFB/PLGA followed by TFB/SLNs. These dates revealed that PLGA-based nanocarriers could directly transport TFB into the brain *via* olfactory pathways (34). Recently, the IN capability of SLNs and PLGA NPs was used for the brain delivery of meloxicam. Meloxicam may have potential effects in controlling AD disease despite its low bioavailability in the brain. The results showed the SLNs demonstrated higher drug loading and encapsulation efficacy than PLGA NPs. The encapsulated meloxicam into the prepared formulations showed enhanced mucoadhesion, permeation, *in vitro* release, bioavailability, and *in vivo* brain distribution (35). The low bioavailability in CNS has been also observed in using rivastigmine, as a reversible dual cholinesterase inhibitor and tolerable drug for AD management. A study evaluated PLGA NPs and nasal liposomal formulations for IN delivery of rivastigmine in acute scopolamine-induced amnesia animal models. As a result, nasal liposomal rivastigmine formulation in comparison with PLGA NPs demonstrated the best pharmacokinetics performance and considerably improved the memory deficit made by colchicine and inhibited acetylcholinesterase (36).

In a study, Huperzine A was trapped on targeted PLGA NPs modified with lactoferrin (Lf)-linked N-trimethylated CS (TMC) (HupA Lf-TMC NPs) for nose-to-brain delivery of HupA for AD management. The formulation has sustained release *in vitro* at 48 hr and drug EE of 73.8%. The formulation showed slight toxicity in the 16HBE cells in comparison with the free drug and the higher cellular uptake exhibited in SH-SY5Y and 16HBE cells. The formulation showed a longer residence time and greater fluorescence intensity in the brain than non-targeted NPs through *in vivo* imaging. Following the IN application of the formulation, the distribution of HupA in the mice’s cerebrum, olfactory bulb, hippocampus, and cerebellum (37). In a study, tacrine-loaded PLGA NPs for AD management *via* IN were developed. The NPs have a low PDI, adequate tacrine EE, and small size. Data from *in vitro* studies revealed a continuous release profile with a maximum release (~ 43.65%) within 120 hr and followed the first-order kinetics. In addition, an *in vivo* study exhibited great brain targeting efficiency through IN application of carbopol 934-based nanocomposite gel of Pt-coated PLGA NPs to rats. Pt-coated NPs compared with uncoated NPs showed higher brain absolute bioavailability of tacrine (Figure 2b) (38). For IN delivery of galantamine, Nanaki and coworkers developed PLGA hybrid NPs and poly (l-lactic acid) (PLLA). The results showed that the NPs have varied sizes of 182 and 394 nm. The results indicated controlled release of galantamine in encapsulated form and successful delivery through the IN route to the hippocampus of male Wistar rats in just a few hours (39).

Studies have shown that an anti-inflammatory and immunosuppressive agent called triptolide, extracted from *Tripterygium wilfordii*, has therapeutic effects on AD. Owing to its poor water solubility, instability, nonspecific biodistribution, and high toxicity, the clinical use of triptolide for AD treatment is limited. Accordingly, triptolide-loaded multiple-coated PLGA NP surfaces were modified with Tween-80, CS hydrochloride, borneol/mentholum eutectic mixture, and PEG for IN drug delivery to the brain. The fabricated formulation was 147.5 nm with an EE of 93.14%, and a loading efficiency of 1.17. In addition, the formulation demonstrated good transcellular permeability to Caco-2 cells *in vitro* and a sustained-release profile. Moreover, the prepared formulations remarkably prevented the enhancement of the intracellular Ca^2+^ influx in PC12 cells caused by Aβ42, decreased oxidative stress, and reduced cytotoxicity (40). Researchers used different sizes of PLGA NPs with size and time-dependent uptake mechanisms in Pig olfactory mucosal layers. The results showed intracellular uptake after 5 min. In addition, in the neuronal fibers, the attendance of PLGA NPs revealed intracellular and transcellular transport by the trigeminal or olfactory nerve in the CNS. Nano-in-Micro particles (NiMPs) include PLGA NPs implanted into CS MPs with reproducible, uniform size distribution, flat surface, and high EE (51%). After 15 min, NiMPs spread to the entire transverse segment of the olfactory mucosa. This faster absorption and wider expansion are related to the use of CS and thus the tight-junction-opening by it. It was observed 15 min after CS particle application, an anti-inflammatory reaction initiated *via* M2-macrophages, because of the beneficial properties of CS including the degree of deacetylation (80%), particle size (0.1–10 µm), and molecular weight (150–300 kDa). Finally, the use of PLGA NPs and NiMPs can deliver drugs to the brain and CNS in three main ways, including intracellular, *via* neurons, paracellular, and transcellular transport (9).

***Parkinson***

A progressive, degenerative CNS disease, which affects the dopaminergic neurons, with severe motor symptoms, including rigidity, postural imbalance, and bradykinesia, is known as Parkinson’s disease (PD). This subsection summarizes the PLGA-based IN drug delivery for PD (Table 2).

Levodopa (L-DOPA) is a prodrug of dopamine that is administered as the first choice and the gold standard in PD treatment. L-DOPA is metabolized to dopamine to treat symptoms of PD (41). The low brain uptake and oral bioavailability due to broad metabolism by aromatic amino acid decarboxylase in the peripheral pathway have limited the use of this drug (42). On the other hand, undesirable L-DOPA conditions with frequent and short dopamine receptor stimulation that lead to dyskinesia and psychic, cardiovascular, and gastrointestinal adverse effects are seen in oral administration (43). It seems biocompatible nano-sized drug carriers could increase bioavailability, therapeutic efficiency, and decrease the unfavorable side effects of the drug (44, 45). 

In a study, the efficacy of L-DOPA, L-DOPA+benserazide, and PLGA-based nano-DOPA through IN administration was compared in the PD model. The results showed that the coordination performance and lasting duration in the nano-DOPA group was significantly higher than in L-DOPA+benserazide and L-DOPA groups. IN nano-DOPA revealed a stable motor function recovery with the effect continued for one week after administration in the 6-OHDA-induced rat PD model. Moreover, PLGA-based NPs had a greater bioavailability, efficacy, and effective half-life (46). Another fabricated L-DOPA-loaded PLGA NPs for nose-to-brain prescription demonstrated prolonged release over about 9 hr and *in vivo *efficacy in the PD mice model (47).

Ropinirole hydrochloride (ROP-HCl) is a non-ergot dopamine D2-agonist, which is used in PD management, but because of extensive hepatic metabolism, has poor oral bioavailability. In order to improve therapeutic efficacy and overcome hepatic first-pass metabolism, ROP-HCl is loaded on PLGA NPs that are surface-modified *via* vitamin E and natural emulsifiers for IN drug delivery. Fabricated formulation demonstrated cumulative drug diffusion of 96.43% in 24 hr, high PS (279.4 nm), EE% (72.3%), and zeta potential (-29.4 MV) (48). In another nasal mucosal delivery of ROP-HCl by PLGA and CS-modified PLGA NPs, the complete release of the drugs in PLGA/CS NPs was verified in a simulated nasal electrolyte solution for 24 hr. PLGA/CS NPs decreased the cell viability of peripheral blood mononuclear cells (PBMC) and Raw 264.7 cells in a concentration-dependent manner. Therefore, PLGA/CS NPs could be a precious vehicle for the ROP-HCl delivery to the CNS (49).

Despite L-DOPA, there are some other therapeutic agents, known as DOPA replacement therapy, that can improve PD symptoms. Regarding this, some researchers used PLGA-based nanocarriers consisting of other effective PD drugs. 

**Figure 3:**
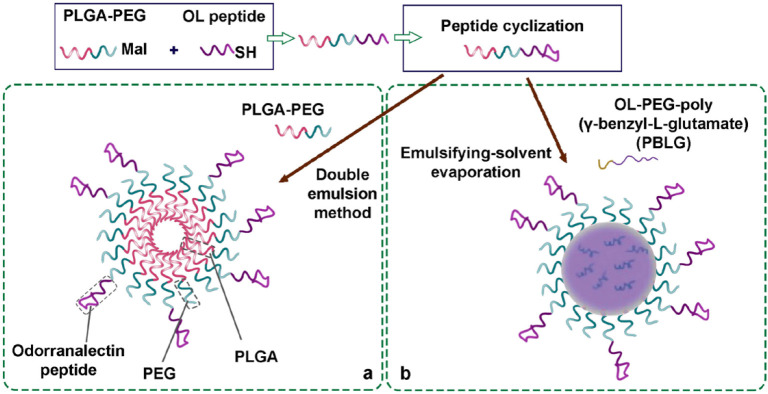
(a&b) Schematic diagram of odorranalectin (OL )-polyethylene glycol (PEG)–PLGA NPs preparation by the double emulsion method using the reaction of maleimide and OL

In a study, Odorranalectin (OL)-loaded PEG-PLGA NPs were used to improve nose-to-brain OL delivery. OL is the smallest lectin with much less immunogenicity (Figure 3a). In addition, urocortin peptide (UCN) was encapsulated into OL-PEG-PLGA NPs to evaluate its therapeutic efficacy in hemi PD rats after IN application by neurotransmitter detection, tyrosine hydroxylase, and rotation behavior assay. The results demonstrated enhanced therapeutic effects of OL-loaded PLGA NPs and improved delivery of NPs in the PD brain (50). In the same study, IN administration of OL-PEG-PLGA NPs was compared with its intravenous injection. The results revealed that IN administration has greater brain targeting efficiency compared with intravenous injection (51). Regarding the OL effect on the elongation of the residence time of CUR in the nasal mucosa and enhancing its cellular uptake, Li *et al.* developed an IN delivery nanocarrier consisting of OL-functionalized CUR-loaded NPs. They formulated CUR into OL-PEG-PLGA conjugate and OL-PEG-poly (γ-benzyl-L-glutamate) (PBLG) to increase the bioavailability, stability, and specificity of CUR (Figure 3b). The results showed that the fabricated formulations have higher efficiency and cellular uptake with no severe toxicity, compared with unmodified NPs. In addition, pharmacokinetic studies demonstrated that the formulation had a higher relative bioavailability (52).

Bi *et al.* reported biodegradable PEG-PLGA NPs with lactoferrin (Lf) surface modification for IN administration of rotigotine for PD management. The pharmaceutical and physicochemical studies demonstrated slow and continuous release of rotigotine from Lf-coated PLGA NPs for 48 hr. Despite the cytotoxic effect of free rotigotine, Lf-coated PLGA NPs did not decrease the viability of SH-SY5Y and 16HBE cells. In addition, the higher brain delivery of rotigotine-loaded Lf-PLGA NPs through IN administration was confirmed by distribution of the drug in the striatum, the primary region affected in PD (53). In another study, the brain delivery potential of rasagiline-encapsulated CS-coated PLGA NPs, through IN administration, was investigated. The double emulsification-solvent evaporation method was employed to prepare CS-coated PLGA NPs. The mean EE, PDI, and particle size were 0.212 ± 0.009, 122.38 ± 3.64, and 75.83 ± 3.76, respectively. Pharmacokinetics investigation in rat plasma and brain revealed outstanding high amplified C_max_ and AUC0-24 over the intravenous treatment group. As a result, after formulation and administration through IN way, significant enhancement of bioavailability in the brain was seen, promising the treatment of PD (54).

***Brain tumor***

**Figure 4:**
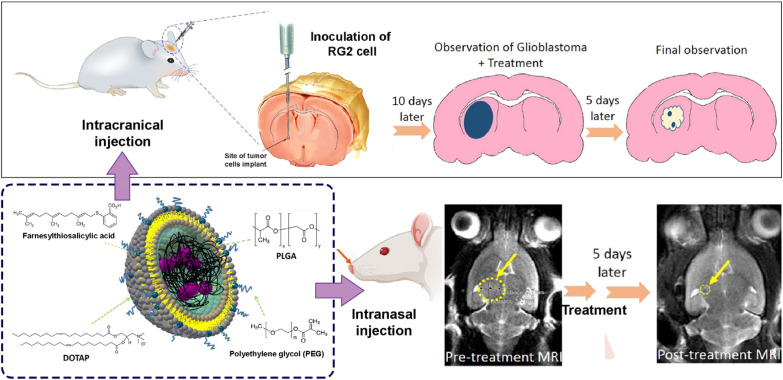
Farnesylthiosalicylic acid (FTA)-loaded polyethylene glycol (PEG)-poly lactic-co-glycolic acid (PLGA) hybrid nanoparticles (NPs) for glioblastoma multiforme (GBM) treatment through intranasal (IN) administration

Glioblastoma multiforme (GBM) is known as an aggressive tumor without any certain cure. In most tumor recurrence cases, after resection, radiation or chemotherapy is needed to postpone the infiltration of the tumor remains (27). In addition, intracerebral chemotherapies were employed to prevent tumor regrowth, while due to weak brain drug distribution, the treatments remain unsuccessful. The NP-based approaches for effective drug delivery through IN pathway provide a new horizon for GBM treatment (Table 3). Researchers developed farnesylthiosalicylic acid-loaded PEG-PLGA hybrid NPs through IN administration to evaluate antitumor efficacy in rats (Figure 4). After 10 days, 5 doses of 500 μM of prepared formulation were inoculated to glioma-bearing rats *via* intravenous and IN pathways. After treatment, formulation exhibited a considerable decrease in tumor area (55). Chu *et al.* designed Ephrin type-A receptor 3 (EPHA3) tyrosine kinase antibodies loaded PLGA NPs for IN-based treatment of Glioblastoma. The results showed sustained release up to 48 hr and cellular uptake by C6 cells. The results of distribution and *in vivo* imaging studies demonstrated effective accumulation in glioma tissues and high fluorescence intensity in the brain confirming the targeting effect of anti-EPHA3. Cell apoptosis significantly increased in anti-EPHA3-loaded NPs. In addition, the anti-EPHA3-modified NPs increased the survival time of flame-bearing rats. The results revealed that this nanocarrier has the potential of IN drug delivery to GBM tumors (56).

**Figure 5:**
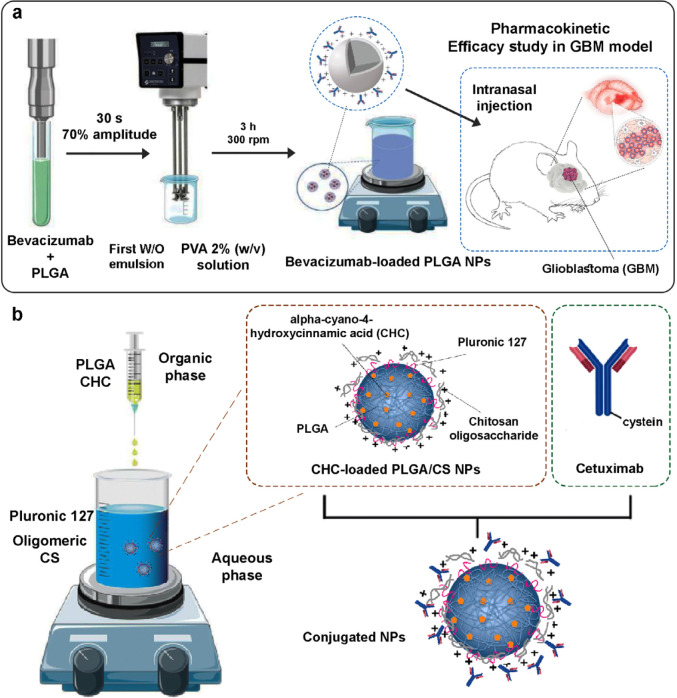
a) Mechanism of nose-to-brain delivery of bevacizumab-loaded poly lactic-co-glycolic acid (PLGA) nanoparticles (NPs), b) Cetuximab-and Cetuximab and alpha-cyano-4-hydroxycinnamic acid (CHCA)- chitosan (CS)/polyethylene glycol (PEG)/poly lactic-co-glycolic acid (PLGA) nanocarriers for intranasal (IN) administration

In recent years, the use of monoclonal antibody considers a new way of GBM treatment. One of the drugs used to treat GBM is Bevacizumab, an anti-angiogenic monoclonal antibody applied to reduce angiogenesis. Recently, bevacizumab-loaded PLGA NPs were developed and intranasally inoculated in CD-1 mice (Figure 5a). After 7 days of administration, the higher brain bioavailability, increased residence time and penetration of bevacizumab-coated PLGA NPs was observed. The results also showed a decrease in tumor growth accompanied by a higher anti-angiogenic effect in comparison with the free drug (57). Researchers designed mucoadhesive NPs based on PLGA NPs modified with CS as a potential platform to co-deliver monoclonal antibody cetuximab and alpha-cyano-4-hydroxycinnamic acid (CHCA) in the nose to brain application (Figure 5b). The formulation and conjugated NPs exhibited cytotoxicity in SW1088 and U251. The antiangiogenic activity of formulation was proved by chicken chorioallantoic membrane assay (58).

**Figure 6:**
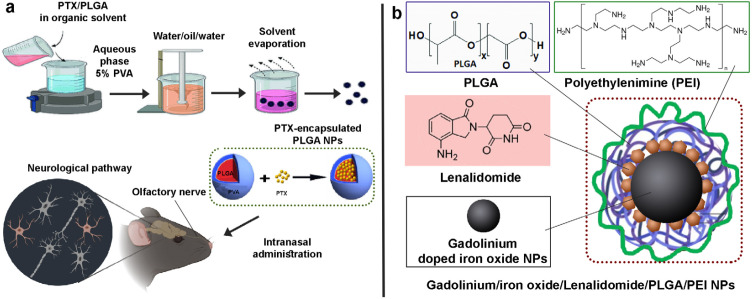
a) Intranasal (IN) delivery of Paclitaxel-encapsulated poly lactic-co-glycolic acid (PLGA), b) Gadolinium/iron oxide/polyethylenimine (PEI)/Lenalidomide/PLGA nanoparticles (NPs) for intranasal (IN) administration

In a study, arginyl-glycyl-aspartic tripeptide (RGD) was conjugated with paclitaxel and loaded on PLGA NPs for IN delivery for the treatment of GBM. The formulations exhibited enhanced anticancer effects *in vivo* and cancer-specific delivery. The results revealed that IN administration effectively reduces the tumor burden by about 75% *via* apoptosis induction and without any effect on the G0 stage of normal cells, inhibiting cancer cell proliferation (59). In another study, IN drug delivery of Paclitaxel encapsulated PLGA NPs to the brain, (Figure 6a) showed a sustained release profile and dose-dependent cytotoxicity to U87MG cells. The biodistribution studies demonstrated high anti-glioblastoma efficacy and *in vivo* uptake through the IN route (60). In a study, synthesized Gadolinium doped iron oxide nanoplatforms and nanoprecipitation technique was used to develop theranostic PLGA NPs and coated Lenalidomide, respectively. In order to provide a target against gliomas, modification of polyethylenimine (PEI) was carried out by targeting moieties, and the cell line studies were done on U87MG glioblastoma cell lines (Figure 6b). The theranostic NPs showed particle size of 122.26 nm and EE of 97.82 %. The results of cell line studies demonstrated the anti-cancer activity of nanoformulation with localization in mitochondria. The results of the toxicity studies revealed that the nanoformulation was non-toxic in comparison with the control group. The potential of the theranostic NPs in brain targeting by IN route has been proved by biodistribution studies (61).

***Ischemia***

Because of the difficulty in drug delivery and the presence of BBB, targeted treatment of cerebral ischemia/reperfusion injury remains challenging (62). Recently, PLGA-based IN drug delivery has been reported as a novel approach to treating ischemia (Table 4). In a study, researchers formulated tanshinone IIA-loaded PLGA NPs with borneol modification to improve IN drug delivery. The results showed a sustained release, EE 3.6%, and an inhibition criteria P-glycoprotein (P-gp). The uptake rate was significantly greater in the targeted group and this uptake was performed using micropinocytosis and clathrin/caveolae-mediated endocytosis. Nanoformulation considerably increased the preventive effect reduced cerebral infarction areas, decreased the content of malondialdehyde on a rat model of CIRI, and enhanced neurological scores through IN administration (63). 

miR-124 can provide a protective effect against cerebral ischemia and successfully modify the symptoms of ischemic brain damage. In a study, RVG29-NPs-miR124 was developed for IN administration to cure neurological injury after cerebral ischemia in middle cerebral artery occlusion (t-MCAO) rat models. The results demonstrated that the neurological and Rhoa scores in RVG29-PEG-PLGA/miRNA-124-received group were considerably lower than in PEG-PLGA/miRNA-124 and RVG29-PLGA/miRNA-124 administrated group. Moreover, the results revealed that the formulation reduced the signs of cerebral ischemia-reperfusion damage (64). In a study, to deliver *via* the IN route in cerebral ischemia-reperfusion, the neuroprotective efficacy of thymoquinone-loaded PLGA-CS NPs has been studied. The prepared formulation considerably enhanced the locomotor activity and grip strength and reduced the ischemia infarct volume in the middle cerebral artery occluded rats (65).

***Epilepsy***

Recently, for the treatment of epilepsy, PLGA-based IN drug delivery has been reported as a novel approach (Table 4).

**Figure 7:**
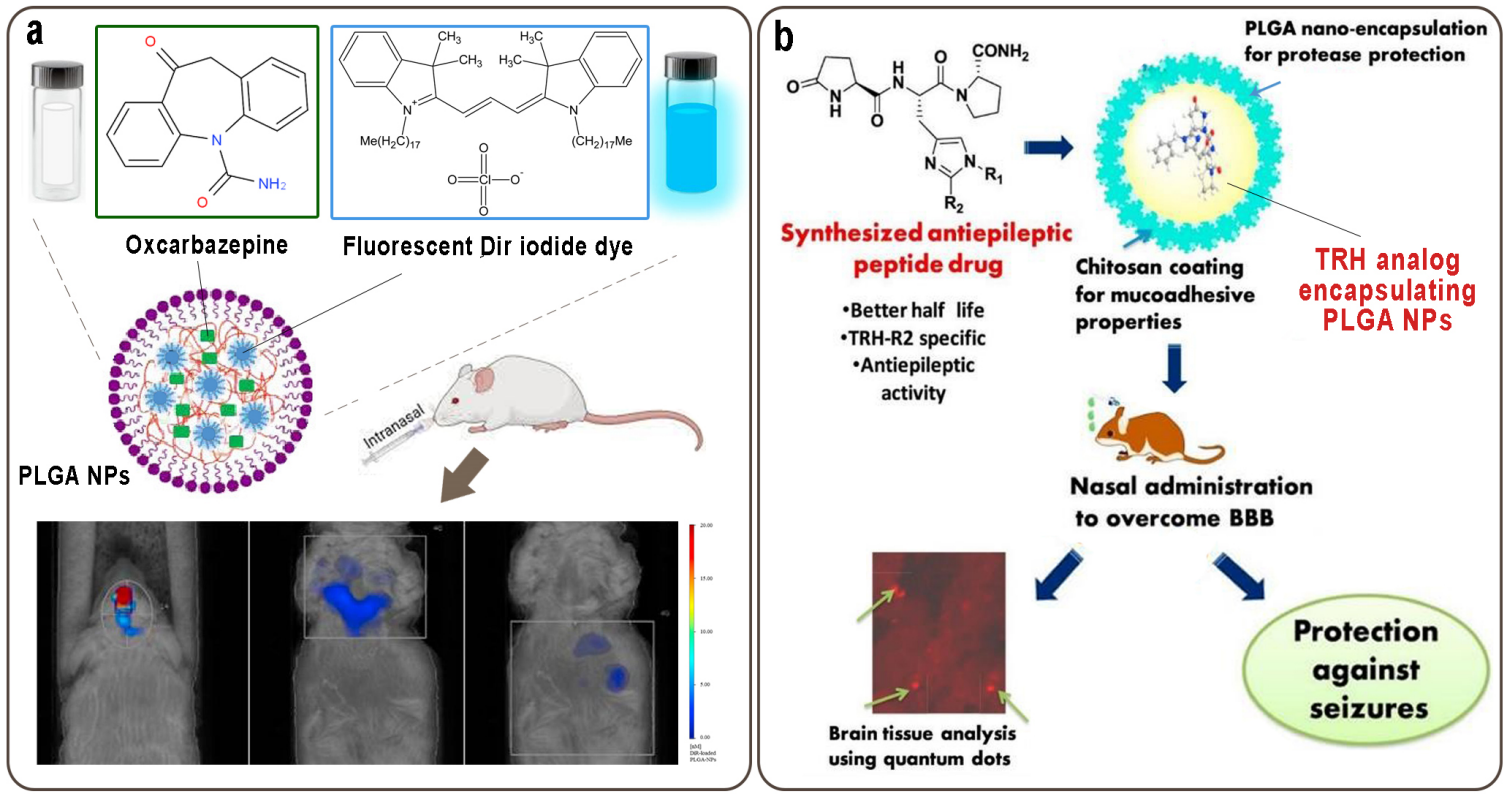
a) IN administration of oxcarbazepine-loaded PLGA NPs along DiR-functionalized PLGA NPs for the targeting of epilepsy, b) Clonazepam-loaded PLGA NPs for seizure treatment via IN delivery

In a study, Oxcarbazepine (OX), an anti-epileptic drug, was entrapped in emulsomes and loaded with PLGA-PEG-PLGA triblock copolymer thermogel. The incorporation of OX emulsomes in thermogels increased its residence time and retarded drug release in rats. The release pattern showed sustained drug release in X-emulsome-thermogel at 53.5% and OX-emulsome at 81.1% for 24 hr. The pharmacokinetic studies revealed transport of OX to the systemic circulation through IN application with the highest MRT for OX-emulsomal-thermogels and higher uptake in the brain tissue in the case of OX-emulsomes in comparison with Trileptal® suspension, OX-solution, and OX-emulsomes (66). Musumeci *et al.* evaluated the potential of OX-loaded PLGA NPs in the improvement of brain-targeting efficiency through IN application (Figure 7a). CSF bioavailability confirmed 0.5 mg/kg OX as an effective dose in controlling epileptic seizures. Fluorescence molecular tomography confirmed the translocation of DiR (1-1′-Dioctadecyl-3,3,3′,3′-tetramethylindotricarbocyanine Iodide)-functionalized PLGA NPs, from the nose to the brain, and the accumulation of NPs in the brain after IN applications. Anti-beta tubulin, anti-caspase3, and anti-neurofilament demonstrated the neuroprotective effect of nanoformulation (67).

Lamotrigine (LTG) is an anti-epileptic medication (also called an anticonvulsant) that is used for the management and treatment of neuropathic pain. In a study, LTG-PLGA NPs were loaded with EE% of 84.87 in LTG-PLGA-NPs for IN delivery. The result of dissolution profiles in simulated cerebrospinal and nasal fluid revealed complete release within 5 hr in CSF. MTT test showed a dose-dependent cytotoxicity pattern for optimized LTG-PLGA-NPs. In addition, cytokine analysis exhibits positive effects of LTG-PLGA-NPs as pro-inflammatory cytokine suppressors. Gamma scintigraphy studies were employed for *in vivo* studies for radiolabeled formulation and drug (99mTc-LTG-PLGA-NPs and 99m Tc-LTG-aqueous) using rats for comparison between intravenous, oral, and IN administrations. The results showed drug target organ transport with 22.81% and drug targeting efficiency with 129.81% in the brain (68). In another study, targeting the efficacy of LTG-conjugated PLGA NPs through the IN application was investigated. The formulation showed particle size of 170.0 nm, EE 71%, zeta potential of -16.6 MV, and PDI of 0.191. The results indicated the biphasic release pattern of the formulation. Gamma scintigraphy and biodistribution studies revealed higher levels of LTG in the brain through the IN application of LTG-PLGA NPs compared with LTG-SOL. IN administration of formulation delayed seizure onset time, induced higher bioavailability, and prolonged release (69).

To enhance the brain bioavailability of catechin hydrate, the IN-administrated CS-coated PLGA NPs exhibited excellent mucoadhesive nature. Pharmacokinetics results indicated enhanced C_max_ with AUC 0–24 in Wistar rat’s brain. In addition, the fabricated formulation has a significantly higher treatment seizure threshold in rodent models. It seems the formulation could increase brain bioavailability in the treatment of epilepsy (70). L-pGlu-(2-propyl)-L-His-L-ProNH2 (NP-647) and L-pGlu-(1-benzyl)-L-His-L-ProNH2 (NP-355) (analogs of thyrotropin-releasing hormone)-loaded PLGA NPs with surface modified CS was developed for IN delivery for the possible antiepileptic properties. The result suggested the effective delivery of neurological drugs to the brain (71). In another work, UHPLC-PDA was used to develop PLGA-NPs and to enhance brain bioavailability for thymoquinone (THQ) from IN application. The emulsion solvent evaporation method was employed to prepare THQ-PLGA-NPs. The formulation exhibited a sustained release pattern and enhanced THQ brain bioavailability through IN administration, compared with intravenous. Furthermore, the formulation improved the seizure threshold treatment (72). In a study, clonazepam was loaded onto PLGA NPs to find out its effect on seizure *via* IN delivery (Figure 7b). The NPs have a particle size of 165.1 nm, and EE of 63.7%. Higher AUC values in the brain and brain/blood uptake ratios following the IN application of formulation revealed brain biodistribution and more effective brain targeting of clonazepam in BALB/c mice. Moreover, the onset of seizures induced by PTZ in rats was considerably delayed by IN delivery of NPs in comparison with intravenous and IN CLO-SOL (73).

***Depression***

The secondary common cause of disability with a debilitating psychiatric condition is known as depression. The main mechanism of action of antidepressants is inhibition of neuronal reuptake of enhanced neurotransmitters. However, the use of these drugs is limited because they failed to reach the brain through the usual ways. It seems PLGA-based nanocarriers can be useful in this issue (Table 4). In a study, Desvenlafaxine-loaded PLGA-CS NPs were prepared by a solvent emulsion evaporation method to investigate the antidepressant efficacy of the formulation from IN delivery. The prepared formulation considerably improved the level of monoamines in the brain and decreased the symptoms of depression through the IN route compared with orally administered Desvenlafaxine. In addition, IN administration of formulation improved the pharmacokinetic profile of Desvenlafaxine (74).

In another work, Venlafaxine-loaded PLGA NPs modified with two ligands against transferrin receptors have been optimized to improve brain delivery of Venlafaxine. In addition, C57/bl6 mice were used for *in vivo* biodistribution studies. The result of the permeability in the hCMEC/D3 cell line revealed that loaded Venlafaxine was not influenced by P-gP pump efflux, enhancing its concentration in the basolateral side after 24 hr. The *in vivo* studies in C57/bl6 mice confirmed that after 30 min, 25% of plain NPs reached the brain while less than 5% of functionalized NPs get the target. It can be concluded that functionalized NPs access the brain *via* receptor-mediated endocytosis while plain NPs probably travel *via* direct IN way (75).

***Schizophrenia***

Schizophrenia is a serious psychic disorder in which people explain reality abnormally. Schizophrenia is characterized by continuous hallucinations, delusions, paranoia, and disorganized thinking that disable daily functioning. The anti-schizophrenia drugs are suitable candidates that can be assayed by PLGA-based IN delivery (Table 4). Regarding Olanzapine (OZ)-loaded PLGA NPs for direct IN delivery to provide sustained release and brain targeting, OZ helps to manage symptoms of schizophrenia and bipolar disorder. The NPs were evaluated for *ex vivo* diffusion, *in vitro* release, pharmacokinetics, and toxicity studies. A biphasic profile with initial burst release followed by sustained release, through Fickian diffusion-based release mechanism, was seen in the drug release study. *Ex vivo* diffusion displayed ~13% drug diffusion in 3.5 hr from NP, through sheep nasal mucosa. No considerable side effects of OZ-loaded NPs were observed in the histopathological study through the sheep nasal mucosa. The brain uptake of OZ-PLGA NPs *via* IN route was 10.86 (76).

To construct a biodegradable nose-to-brain drug delivery system, STL-conjugated PLGA NPs (STL/PLGA NPs) were optimized. The result of *in vitro* uptake study revealed an increase in endocytosis of STL/PLGA NPs in comparison with unmodified PLGA NPs in Calu-3 cells. Coumarin-6 was rapidly absorbed into the brain and blood following the IN application. The AUC of coumarin-6 in the olfactory bulb, cerebellum, cerebrum, and blood were about 1.48, 1.45, 1.89, and 0.77-fold of those of free NPs, respectively. STL/PLGA NPs exhibited greater brain targeting efficacy in different brain tissues, which was confirmed by a near-infrared fluorescence probe image. Negligible cilia irritation and mild cytotoxicity of STL/PLGA NPs were indicated by safety experiments (77). In a work by Piazza *et al.,* haloperidol-loaded lectin-functionalized, PEG–PLGA NPs were developed for schizophrenia treatment. NPs showed high encapsulation efficiencies, drug-loading capacities, and STL conjugation efficiencies. After 96 hr, the *in vitro* haloperidol release was 6–8%, revealing minimal drug leakage and effective drug carrying to the targeted site in the brain. The released drug successfully bound to bovine striatal dopamine D2 receptors and displaced [3H] N-propylnorapomorphine, suggesting the inducing catalepsy. Following the IN application of the formulation, haloperidol concentrations increased in the brain (78). Another STL/PLGA-based nanocarrier for schizophrenia treatment through IN delivery was validated using haloperidol-loaded STL-functionalized PEG-PLGA. The result indicated that IN application of formulation increased brain tissue haloperidol concentrations in comparison with STL-functionalized NPs administered IN with a pipette (79).

***Conclusions and future perspectives***

Because of the stringency of BBB, drug targeting of CNS diseases is challenging. Therefore, oral and parenteral administration reduces the efficacy and potency of drugs. The nose-to-brain drug delivery is an alternative pathway for the treatment of brain diseases. IN route bypasses the BBB and transports the drug directly to the brain from the nasal cavity. Various research claims that IN-based brain drug delivery overcomes the limitations of other drug delivery processes. However, there are some drawbacks related to IN delivery such as poor drug permeation *via* the nasal mucosa, mucociliary clearance, lack of control over the dose of the drug spray, lack of uniform pressure, and duration of the spray. Recently, the NPs-based drug delivery system is considered a promising option to improve IN drug delivery to the brain. 

Studies have shown that PLGA with good biocompatibility and biodegradability has attracted a lot of interest from researchers. PLGA is a good coating material. It is often used to improve the solubility and stability of hydrophobic therapeutic agents. In addition, PLGA is a safe FDA-approved copolymer. These phenomena lead to using of PLGA-based nanocarriers for IN delivery. As abovementioned, PLGA-based nanocarriers have been successfully used to target neurodegenerative diseases for example AD, PD, and other brain disorders such as brain tumors, ischemia, epilepsy, depression, and Schizophrenia through IN delivery. However, these nanocarriers are currently in the nascent phase. 

Despite some advantages, some limitations can affect PLGA-based nose-to-brain drug delivery. The small volume (~100 μl) of nanocarriers in IN administration, the short retention time for drug absorption, the influence of mucosal secretion, and the irritation and allergic effects are the main shortcomings of this process. Furthermore, the PLGA-based formulations for nasal drug delivery cannot be used in a liquid form.

Future directions for IN PLGA-based drug delivery include improving delivery strategies by using specific targeting units such as aptamers or peptides, optimizing the concentrations of therapeutic agents, and using permeation-enhancing agents in the formulation of PLGA-based nasal drug delivery nanocarriers. An approach to enhance nasal adsorption of NPs is surface modification. CS could be a good nominee as a shell material for the surface modification of PLGA-based nanoformulations. The positive charge of CS helps nanocarriers electrostatically interact with negatively charged receptors such as mucin. It has been demonstrated that CS can enhance the residence time of NPs at the target site and play a role in opening tight junctions and improve drug permeation *via* nasal mucosa. Lectins are other biorecognition ligands, which can be used as targeting units in PLGA nano vehicles due to specific and reversible binding to oligosaccharides in the target site. In addition, the aptamer is another ligand for this issue.

Recently, aptamer-based drug delivery has attracted special attention for accessible and specific treatment of diseases. To date, there has been no report about PLGA-based IN administrated nano-drug, which could be employed clinically. Given the progress of aptamer-based devices in clinical application, the aptamer-targeted PLGA loaded with monoclonal antibodies can be endorsed as a novel useful progression platform for PLGA-based IN therapy in the future. 

IN: Intranasal

**Table 1:** Poly lactic-co-glycolic acid (PLGA)-based nanocarriers for intranasal (IN) delivery of anti-alzheimer’s disease (AD) drugs

**Drug**	**Nanocarrier formulation**	**Mechanism of action**	**Ref.**
Curcumin (CUR)	CUR/PLGA CUR/PLGA/Tet-1 peptide	- Anti-oxidant - Destroy Aβ	(28)
	CUR/PLGA Bisdemethoxy-CUR/PLGA	- Interaction with mucin, cyclooxygenase2, and Aβ masses	(29)
	CUR/HP-β-CD CUR-CS-PLGA	- Antioxidant - Decrease of IL-6 and TNF-α with anti-inflammatory effects	(30)
	CUR/CS PLGA	- Antioxidant - Caveolae-mediated endocytosis	(31)
Insulin	CPPs/PLGA	- Enhanced penetration into the brain	(32)
Lectins (STL)	bFGF/STL/PEG-PLGA	- Bind to N-acetylglucosamine	(33)
Tarenflurbil (Flurizan/TFB)	TFB-PLGA TFB/SLNs	- Enhanced penetration into the brain	(34)
Meloxicam (Mel)	Mel/PLGA Mel/SLNs	- Enhanced mucoadhesion, permeation	(35)
Huperzine A (HupA)	HupA/Lf/TM-CS/PLGA	- Targeted brain distribution	(37)
Tacrine	Tacrine/PLGA carbopol 934/Pt/PLGA	- Enhanced bioavailability	(38)
Galantamine	Galantamine/PLGA Galantamine/PLLA	- Controlled release of galantamine - Successful delivery to the hippocampus	(39)
Triptolide	Triptolide/PLGA/CS	- Prevention the intracellular Ca^2+^ influx caused by Aβ42	(40)
NiMPs	PLGA/CS NiMPs	- Anti-inflammatory	(9)
Rivastigmine	Rivastigmine/PLGA Rivastigmine/liposome	- Improve the memory deficit - Acetylcholinesterase inhibition	(36)

IN: Intranasal; CUR: Curcumin; PLGA: Poly lactic-co-glycolic acid; CS: Chitosan; AD: Alzheimer’s disease

**Table 2:** Poly lactic-co-glycolic acid (PLGA)-based nanocarriers for intranasal (IN) delivery of anti-parkinson’s disease (PD) drugs

**Drug**	**Nanocarrier formulation**	**Mechanism of action**	**Ref.**
Levodopa (L-DOPA)	L-DOPA/benserazide L-DOPA/PLGA	- Dopaminergic effect	(46)
	L-DOPA/PLGA		(47)
Ropinirole hydrochloride (ROP-HCl)	ROP-HCl/PLGA	- Dopaminergic effect	(48)
	ROP-HCl/CS/PLGA	- Dopaminergic effect - ROS	(49)
Odorranalectin (OL)	OL/PEG-PLGA UCN/OL/PEG-PLGA	- Enhanced the neurotransmitter activity - Targeted L-fucose on the olfactory epithelium	(50)
	OL/PEG-PLGA		(51)
CUR/OL	CUR/OL/PEG-PLGA CUR/OL/PBLG	- Targeted L-fucose on the olfactory epithelium Anti-oxidant	(52)
Rotigotine	Rotigotine/Lf/PEG-PLGA	- Continuous release of rotigotine	(53)
Rasagiline	Rasagiline/CS/PLGA	- Enhanced mucoadhesion, permeation	(54)

**Table 3:** Poly lactic-co-glycolic acid (PLGA)-based nanocarriers for intranasal (IN) delivery of brain tumor drugs

**Drug**	**Nanocarrier formulation**	**Mechanism of action**	**Ref.**
Farnesylthiosalicylic acid	Farnesylthiosalicylic acid/PEG-PLGA	- Targeted-therapy of GBM	(55)
Ephrin type-A receptor 3 (EPHA3) tyrosine kinase antibodies	Anti-EPHA3/PLGA	- Anti-EPHA3 targeted GBM treatment	(56)
Bevacizumab	Bevacizumab/PLGA	- Anti-angiogenic monoclonal antibody for GBM	(57)
Cetuximab and alpha-cyano-4-hydroxycinnamic acid (CHCA)	Cetuximab and CHCA/CS/PLGA	- Antiangiogenic activity at GBM site	(58)
Paclitaxel	RGD/paclitaxel/PLGA	Enhanced anticancer effects Apoptosis induction in GBM cells	(59)
	paclitaxel/PLGA	- Sustained release - Anti-glioblastoma efficacy	(60)
Lenalidomide	Gadolinium/iron oxide/PEI/Lenalidomide/PLGA/	- Theranostic NPs	(61)

**Table 4:** Poly lactic-co-glycolic acid **(**PLGA)-based nanocarriers for intranasal (IN) delivery of drugs for ischemia, epilepsy, depression, and epilepsy

**Disease **	**Drug**	**Nanocarrier formulation**	**Mechanism of action**	**Ref.**
Ischemia	Tanshinone IIA	Tanshinone IIA/PLGA/borneol	- Inhibition of P-glycoprotein (P-gp) - Micropinocytosis and clathrin/caveolae-mediated endocytosis	(63)
	miR124	RVG29-PEG-PLGA/miRNA-124	- Reduced the signs of cerebral ischemia-reperfusion damage	(64)
	Thymoquinone	Thymoquinone/PLGA-CS	- Enhanced locomotor activity and grip strength	(65)
Epilepsy	Oxcarbazepine (OX)	OX/PLGA-PEG-PLGA triblock	- Anti-inflammatory - Neuroprotective effect	(66)
		OX/PLGA		(67)
	Lamotrigine (LTG)	LTG/PLGA	- Sustained release - Pro-inflammatory cytokine suppressors	(68)
			- Induced higher bioavailability	(69)
	Catechin hydrate	Catechin/CS/PLGA	Excellent mucoadhesive nature	(70)
	NP-647 and NP-355 analogs of thyrotropin	NP-647&NP-355/PLGA/CS	- Sustained-release properties	(71)
	Thymoquinone (THQ)	UHPLC-PDA/PLGA/THQ	- Sustained release pattern	(72)
	Clonazepam	Clonazepam/PLGA	- Brain biodistribution	(73)
Depression	Desvenlafaxine	Desvenlafaxine/ PLGA-CS	- Improve the level of monoamines in the brain and decrease the symptoms of depression	(74)
	Venlafaxine	Venlafaxine/PLGA	- Targeted delivery based on transferrin receptors	(75)
Schizophrenia	Olanzapine (OZ)	OZ/PLGA	- Sustained release pattern	(76)
	STL/PLGA	STL/PLGA	- Receptor-mediated endocytosis	(77)
	Haloperidol	Haloperidol/STL/PEG-PLGA	- Bound to bovine striatal dopamine D2 receptors and displaced the [3H] N-propylnorapomorphine	(78) (79)

IN: Intranasal; PLGA: Poly lactic-co-glycolic acid; NPs: Nanoparticles; DiR: 1-1′-Dioctadecyl-3,3,3′,3′-tetramethylindotricarbocyanine Iodide

## Authors’ Contributions

N AD and R Kh helped with writing and wrote the original draft. B GN, M Sh, and MS R contributed to writing, review, and editing. M R and Z S conceived and designed the study, and contributed to writing and critical revision.

## Conflicts of Interest

The authors declare no competing interests.
